# Jellyfish as an Alternative Source of Bioactive Antiproliferative Compounds

**DOI:** 10.3390/md20060350

**Published:** 2022-05-25

**Authors:** Gennaro Riccio, Kevin A. Martinez, Jesús Martín, Fernando Reyes, Isabella D’Ambra, Chiara Lauritano

**Affiliations:** 1 Department of Ecosustainable Marine Biotechnology, Stazione Zoologica Anton Dohrn, Villa Comunale, 80121 Napoli, Italy; gennaro.riccio@szn.it; 2 Fundación MEDINA, Centro de Excelencia en Investigación de Medicamentos Innovadores en Andalucía, Avda. del Conocimiento 34, 18016 Granada, Spain; kevin.martinez@medinaandalucia.es (K.A.M.); jesus.martin@medinaandalucia.es (J.M.); fernando.reyes@medinaandalucia.es (F.R.); 3 Department of Integrative Marine Ecology, Stazione Zoologica Anton Dohrn, Villa Comunale, 80121 Napoli, Italy; isabella.dambra@szn.it

**Keywords:** scyphomedusae, cubomedusae, drug discovery, antiproliferative, apoptosis, melanoma

## Abstract

Jellyfish are commonly considered a nuisance for their negative effects on human activities (e.g., fisheries, power plants and tourism) and human health. However, jellyfish provide several benefits to humans and are commonly eaten in eastern countries. Additionally, recent studies have suggested that jellyfish may become a source of high-value molecules. In this study, we tested the effects of the methanolic extracts and enriched fractions, obtained by solid-phase extraction fractionation, from the scyphomedusae *Pelagia noctiluca*, *Rhizostoma pulmo*, *Cotylorhiza tuberculata* and the cubomedusa *Caryddea marsupialis* on different human cancer cell lines in order to evaluate a potential antiproliferative activity. Our results indicated that fraction C from *Caryddea marsupialis*-(CM) and *C. tuberculata* oral arms (CTOA) were the most active to reduce cell viability in a dose-dependent manner. LC/MS based dereplication analyses highlighted that both bioactive fractions contained mainly fatty acids and derivatives, with CM additionally containing small peptides (0.7–0.8 kDa), which might contribute to its higher biological activity. The mechanism of action behind the most active fraction was investigated using PCR arrays. Results showed that the fraction C of CM can reduce the expression of genes involved in apoptosis inhibition in melanoma-treated cells, which makes jellyfish a potential new source of antiproliferative drugs to be exploited in the future.

## 1. Introduction

The marine environment and the organisms living within it are being increasingly exploited as a source of natural defense metabolites and bioactive compounds [1,2,3]. Within marine organisms, jellyfish, particularly scyphomedusae, are raising increasing interest as a source of compounds for biotechnological applications [4,5,6]. Generally considered a nuisance, particularly during their sudden and massive appearances (blooms), because they interfere with human activities at sea along the coasts (e.g., tourism, fisheries and industries) by stinging swimmers, damaging fishing gears and caught fish as well as clogging power plant water inflows [7], jellyfish have been an important part of the diet of eastern populations, particularly the Chinese [8]. Despite the lack of specific studies, scyphomedusae appear to be a healthy food for their low carbohydrate and lipid content and, conversely, their high protein content [4]. In addition to the biochemical composition, recent studies have highlighted the antioxidant activity of several scyphomedusae, including *Cotylorhiza tuberculata*, *Rhizostoma pulmo*, *R. luteum*, *Catostylus tagi* and *Rhopilema esculentum* [9,10,11,12,13], which makes them a healthy food [14] and a source of antioxidant compounds [13].

In addition to the exploitation for antioxidant compounds, jellyfish possess collagen, which is the polymer most abundant in this group. Collagen extracted from jellyfish has shown a high biocompatibility with human collagen [15,16], which prompted biomedical applications, including a substrate for microglia culture [15] and scaffolds for tissue growth and regeneration [17,18]). Although the venom extracted from several jellyfish causes painful stings to humans, it has shown effects to be considered a promising tool for pharmacological products [5]. For example, the venom extracted from *Nemopilema nomurai* was tested on heart and muscle myoblasts in mice and blood cells from different organisms, including humans [19] and a model animal [20], as well as human hepatocellular carcinoma (HepG2) cells [21]. However, the information necessary to develop the application of compounds extracted from jellyfish for biotechnology, particularly biomedicals, is still very limited.

The scyphomedusae *Pelagia noctiluca* (Forsskål, 1775), *Rhizostoma pulmo* (Macri, 1778) and *Cotylorhiza tuberculata* (Macri, 1778) are endemic and often blooming in the Mediterranean Sea [22,23,24,25,26]. *P. noctiluca* is well known not only for its blooms, but also for stinging swimmers in coastal areas along the whole Mediterranean since ancient times [22,23,24,25]. The venom extracted from the scyphomedusa has shown antiproliferative as well as cytolytic and cytotoxic activities [27,28], and induces oxidative stress [29]. Additionally, analgesic [30] as well as anti-inflammatory activities have been detected in the crude venom extracted from *P. noctiluca*.

*R. pulmo* and *C. tuberculata* occasionally bloom in the Mediterranean Sea [22,24,25,26], but they are being studied for their antioxidant activity and their biochemical composition, for which they have been suggested to become a novel food for Mediterranean populations [14]. Because these scyphomedusae induce mild stings (*R. pulmo*) or are completely harmless to humans (*C. tuberculata*), their venom has been less studied than the crude venom of other scyphomedusae [31]. Nevertheless, in vitro bioassays have indicated that the tissue of *R. pulmo* have cytolytic and hemolytic activities [32]. A metalloproteinase, named rhizoproteinase, was isolated from the tentacles of this species and showed anticoagulant activity [33], while Cariello and co-workers isolated rhizolysin, a high-molecular-weight protein with hemolytic activity [34]. The intermediate phase (IP) of the hydro alcoholic extract of *C. tuberculata* has shown antiproliferative activity on MCF-7 breast cancer cell viability [35]. In particular, IP showed a concentration dependent activity, with no effects on non-malignant human epidermal keratinocytes (HEKa).

The cubomedusa *Carybdaea marsupialis* is found typically in the Mediterranean Sea [36]. Recorded in the Adriatic Sea for the first time in 1985 [37] and in Tunisia in 2015 [38], it appears in aggregations of solitary individuals [39]. The apparent increase in the individuals belonging to this species has been attributed to a greater availability of substrate for polyps [22]. Like most cubomedusae, *C. marsupialis* induces painful stings to humans, which become lethal only in rare cases. A pore-forming toxin was extracted from *C. marsupialis* collected in the Adriatic Sea [40], Sanchez-Rodrigues and collaborators isolated one neurotoxin and three cytolysins from specimens identified as *C. marsupialis* in the Caribbean Sea and tested their effect in vivo on sea crabs [41]. The presence of venom-derived neurotoxins that act on membrane proteins of the vertebrate nervous system was corroborated by bioassays on oocytes expressing membrane proteins from rat brain cells [42].

In this study, we tested the effect of total extracts and fractions from the scyphomedusae *P. noctiluca*, *R. pulmo*, *C. tuberculata* and the cubomedusa *C. marsupialis* on different human tumor cell lines (i.e., human hepatocellular liver carcinoma, melanoma and alveolar basal epithelial adenocarcinoma) in order to evaluate their possible antiproliferative activities. Our results indicated that raw extracts and the fraction enriched in glycolipids and phospholipids of *C. tuberculata* (particularly the oral arms) and *C. marsupialis* were the most active and their mechanism of action was studied using cell-death PCR array.

## 2. Results

### 2.1. Raw Extracts Screening

**Figure 1:**
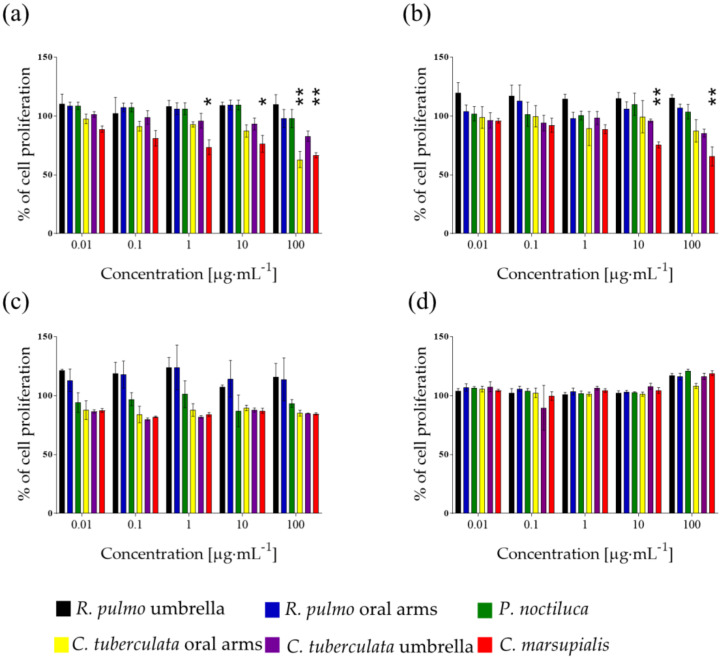
Antiproliferative assay. Raw methanolic extracts were tested at increasing concentration (10 and 100 ng·mL^−1^, 1, 10 and 100 μg·mL^−1^) on A2058 (**a**), A549 (**b**), HepG2 (**c**) and MRC5 (**d**) cell lines. Cell proliferation was normalized using a control sample, containing DMSO only. Results are expressed as a percentage survival after 72 h exposure (*n* = 3; * for *p* < 0.05; ** for *p* < 0.01).

In order to evaluate the antiproliferative activity of the collected jellyfish, raw methanolic extracts of *C. tuberculata* umbrella (CTU), *C. tuberculata* oral arms (CTOA), *C. marsupialis* (CM), *R. pulmo* umbrella (RPU), *R. pulmo* oral arms (RPOA) and *P. noctiluca* (PN) were tested on different human tumor cell lines: hepatocellular liver carcinoma (HepG2), human melanoma cells (A2058), adenocarcinomic human alveolar basal epithelial cells (A549) and human normal lung fibroblasts cell lines (MRC5). Total extract from CM was active on both A2058 and A549 cell lines (Figure 1a,b). In particular, CM was active on A2058 when tested at 1, 10 and 100 µg·mL^−^^1^ (*p* < 0.05, 0.05 and 0.01, respectively), and on A549 at 10 and 100 µg·mL^−^^1^ (Figure 1a,b). CTOA was active only on A2058 at 100 µg·mL^−^^1^ (*p* < 0.01; Figure 1a). Cell proliferation of HepG2 cells and normal cells (MRC5) was not affected by jellyfish total extracts (Figure 1c,d). CTU, RPU, RPOA and PN raw extract did not significantly affect cell proliferation of tested cell lines (Figure 1a–d).

### 2.2. Bioactivity Testing of CTOA and CM Fractions

**Figure 2:**
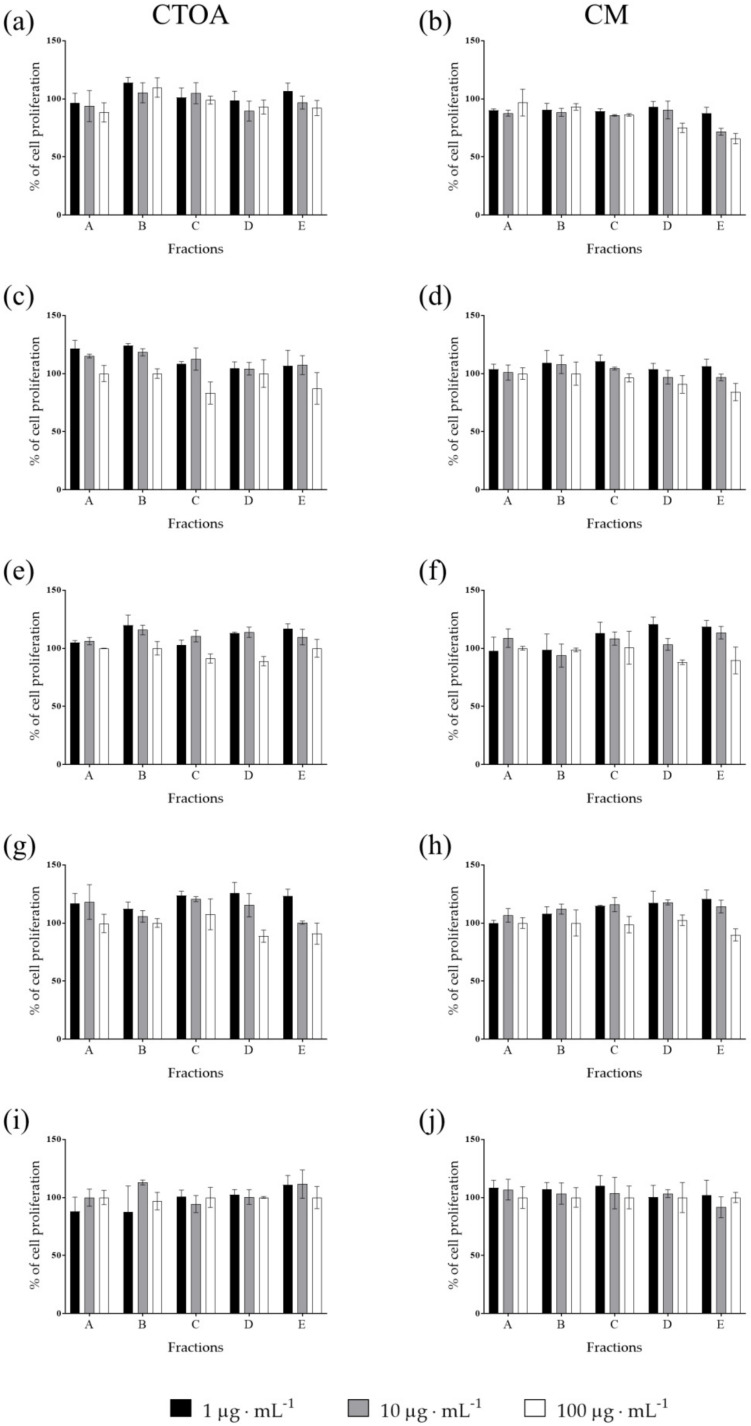
Antiproliferative assay. Fractions obtained from raw extracts of CTOA and CM were tested at increasing concentration (1, 10 and 100 μg·mL^−1^) on A2058 (**a**,**b**), A549 (**c**,**d**), HepG2 (**e**,**f**), HaCaT (**g**,**h**) and MRC5 (**i**,**j**) cell lines. Cell proliferation was normalized using a control sample, containing only DMSO. Results are expressed as percent of cell proliferation after 24 h exposure.

**Figure 3:**
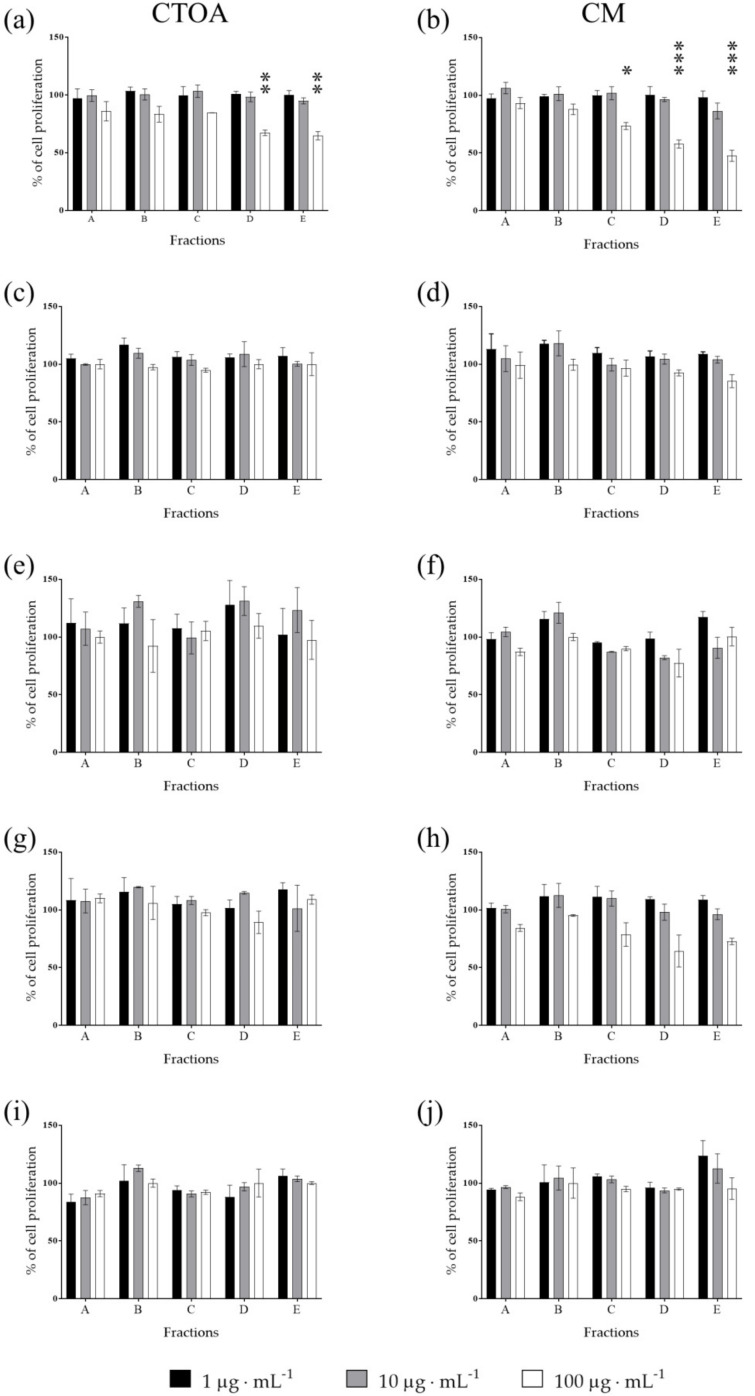
Antiproliferative assay. Fractions obtained from raw extracts of CTOA and CM were tested at increasing concentration (1, 10 and 100 μg·mL^−1^) on A2058 (**a**,**b**), A549 (**c**,**d**), HepG2 (**e**,**f**), HaCaT (**g**,**h**) and MRC5 (**i**,**j**) cell lines. Cell proliferation was normalized using a control sample, containing only DMSO. Results are expressed as percent of cell proliferation after 48 h exposure (*n* = 3; * for *p* < 0.05; ** for *p* < 0.01; ***p < 0.001).

**Figure 4:**
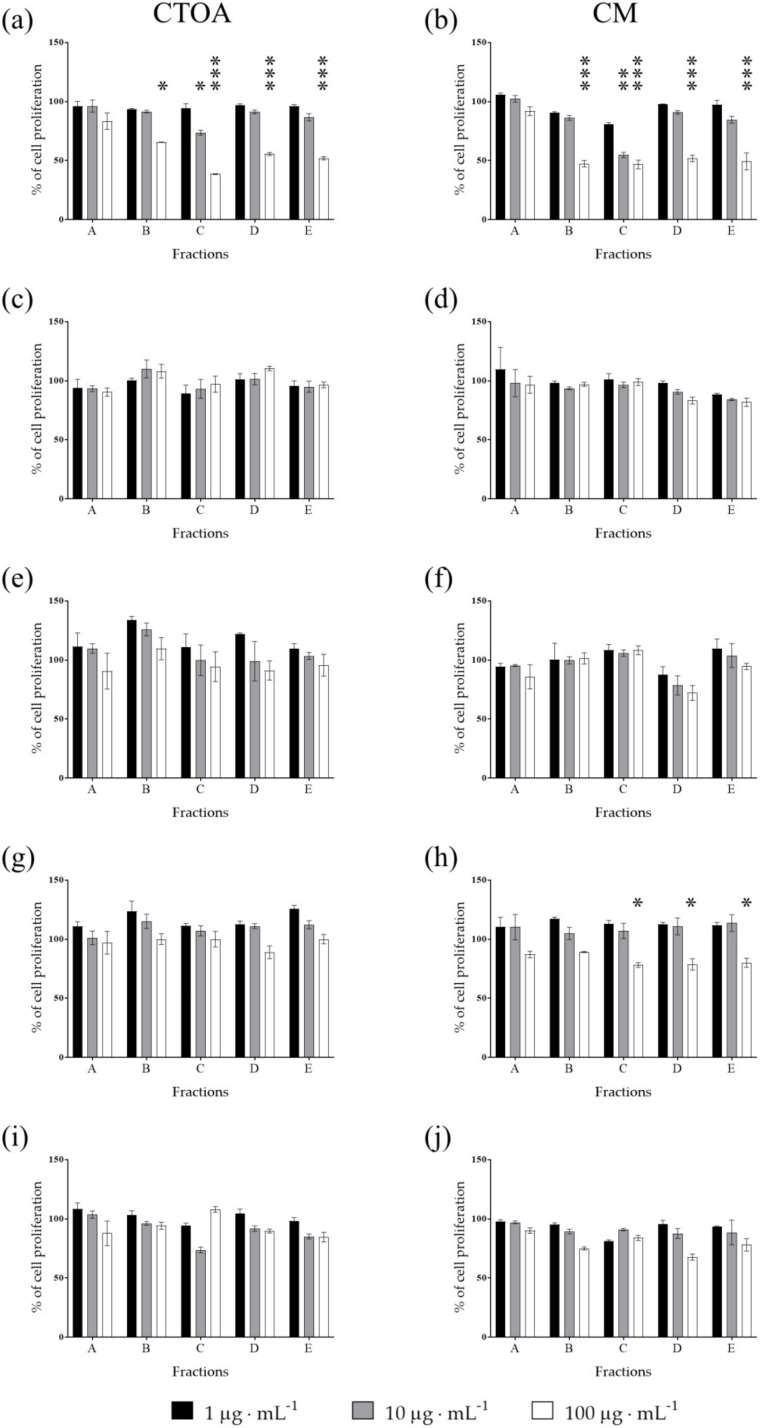
Antiproliferative assay. Fractions obtained from raw extracts of CTOA and CM were tested at increasing concentration (1, 10 and 100 μg·mL^−^^1^) on A2058 (**a**,**b**), A549 (**c**,**d**), HepG2 (**e**,**f**), HaCaT (**g**,**h**) and MRC5 (**i**,**j**) cell lines. Cell proliferation was normalized using a control sample, containing only DMSO. Results are expressed as percent of cell proliferation after 72 h exposure (*n* = 3; * for *p* < 0.05; ** for *p* < 0.01; ***p < 0.001).

Raw extracts of the most active jellyfish (CTOA and CM) were fractioned using a Chromabond SPE column (see methods) and fractions (A-E) were then tested in order to identify the most active ones at 24, 48 and 72 h (Figure 2, Figure 3 and Figure 4). After 72 h of treatment, fraction C of both CTOA and CM showed the highest activity, both at 10 µg·mL^−^^1^ and 100 µg·mL^−^^1^ against A2058 cells (*p* < 0.05 and 0.001 for CTOA 10 µg·mL^−^^1^ and 100 µg·mL^−^^1^, and *p* < 0.01 and 0.001 for CM 10 µg·mL^−^^1^ and 100 µg·mL^−^^1^). Fractions B, D and E of both CTOA and CM were active at 100 µg·mL^−^^1^ on A2058 cells (*p* < 0.05 for CTOA fraction B and *p* < 0.001 for CTOA fraction D and E, while *p* < 0.01 for CM fractions B, D and E; Figure 4a,b). Conversely, a significant antiproliferative activity was not found against the other cancer cell lines (Figure 4c–j). In addition, after 24 h of treatment (Figure 2), fractions from both CTOA and CM did not show any antiproliferative activity. On the contrary, after 48 h of treatment, fraction D and E at 100 µg·mL^−^^1^ from CTOA and fractions C, D, and E at 100 µg·mL^−^^1^ from CM exerted antiproliferative activity on A2058 cell line (Figure 3), and cell proliferation after 48 h of treatment was higher than the one after 72 h of treatment.

**Figure 5:**
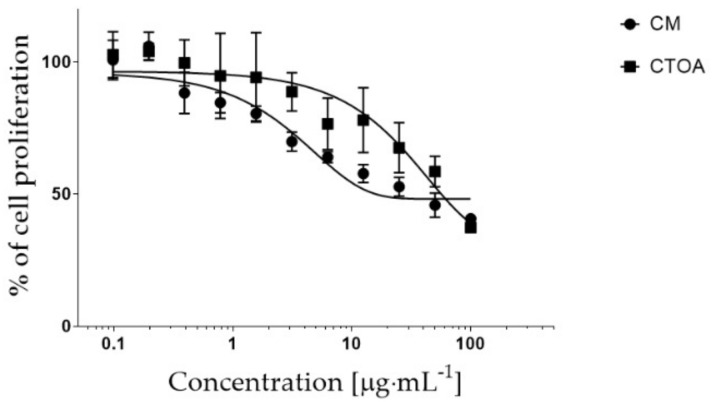
Antiproliferative curve of fraction C of both CM and CTOA. Fraction C was tested at increasing concentrations, from 0.1 to 100 µg·mL^−1^, in A2058 cells. Results are expressed as a percentage of cell proliferation after 72 h exposure (*n* = 3).

In addition, the half-maximal inhibitory concentration (IC_50_) of fraction C was evaluated for both CTOA and CM on A2058 cells (Figure 5). In particular, the corresponding C fractions were tested at increasing concentrations, from 0.1 to 100 µg·mL^−^^1^. Fraction C obtained from CTOA had an IC_50_ = 8.10 µg·mL^−^^1^, while fraction C of CM was the most active, with an IC_50_ = 0.34 µg·mL^−^^1^ (Figure 5).

### 2.3. Mechanism of Action of Fraction C of CTOA and CM

In order to elucidate the cell death metabolic pathway induced by fraction C of the most active jellyfish, expression levels of selected genes involved in various cell death pathways were evaluated using a PCR array in A2058 cells treated in the presence of enriched fraction C of both CTOA and CM (respectively at 20 μg·mL^−^^1^ and 1 μg·mL^−^^1^). Gene transcription was considered to be affected by compounds if expression values were greater than a two-fold difference with respect to the control (DMSO alone; Student’s *t*-test *p* value < 0.05). Both differentially up-regulated and down-regulated genes are reported in Table 1. 

Gene expression analyses indicated that apoptotic peptidase activating factor 1 (APAF1) was up-regulated in both CTOA- and CM-treated cells. APAF1 plays an important role in apoptosis, since it is essential for apoptosome formation. The in vivo loss of APAF-1 expression is generally associated with tumor progression [43] and its up-regulation is associated with the antitumor activity of taxane [44]. S100 calcium binding protein A7A (S100A7), also known as psoriasin, has been found to be up-regulated in CM-treated cells. S110A7 has been found to be up-regulated in psoriatic lesions [45] and when secreted it can be a chemoattractant which regulates the migration of neutrophils or T helper cells [46]. Beclin 1 (BECN1), an autophagy-related gene [47], is over-expressed in CM-treated cells. The DENN/MADD domain containing 4A (DENND4) was down-regulated in both CTOA- and CM-treated cells. DENND4 is a regulator of Rab-GTPase (a class of protein involved in membrane trafficking [48]) [49]. The X-linked inhibitor of apoptosis (XIAP), nucleolar protein 3 (apoptosis repressor with CARD domain, NOL3) and Forkhead box I1 (FOXI1) were down-regulated in CM-treated cells. XIAP, strongly inhibited by CM treatment (−149.92 ± 93.7 fold), is an apoptosis inhibitor; it suppresses apoptosis by binding caspase and inhibiting caspase activation [50]. XIAP modulation is becoming an important target for cancer therapy, in fact, it may confer therapeutic resistance and may modulate signaling factors involved in other processes, such as necroptosis, autophagy and immunosuppression [51]. NOL3 is an apoptosis suppressor and is related to chemoresistance and radiotolerance in cancer cell [52]. FOXI1 is a transcription factor related to cell growth and differentiation and it has been associated with metastasis in breast cancer [53]. Altogether, gene expression analyses suggested an apoptotic signal by APAF1 increase in both CTOA- and CM-treated cells, and also decrease in the apoptosis repressors XIAP and NOL3 in CM-treated cells.

### 2.4. HPLC-UV-HRMS Dereplication of the Fractions

**Figure 6:**
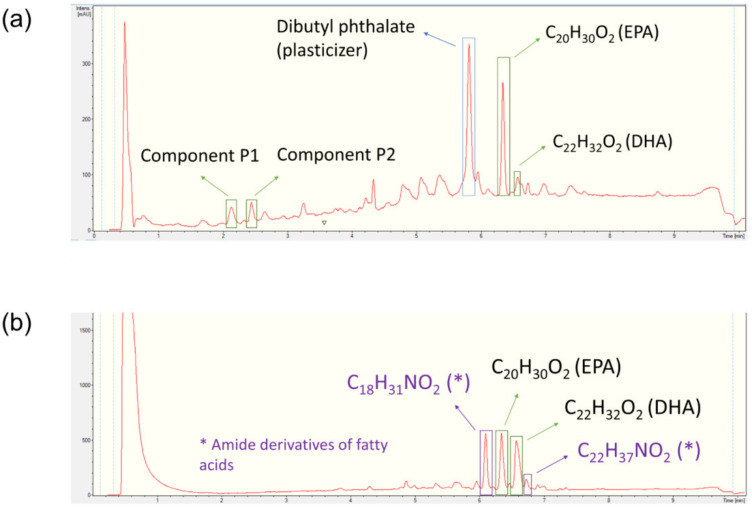
LC-UV chromatogram of fraction C from CM (**a**) and CTOA (**b**).

The bioactive fractions from the raw extracts CM and CTOA were dereplicated by HPLC-UV-HRESIMS according to the procedures reported in the Materials and Methods section. LC-UV chromatograms of fractions C from CM and CTOA are reported in the figures below together with the molecular formula and the putative identity of the most representative components detected by HRESIMS (Figure 6a,b). 

**Figure 7:**
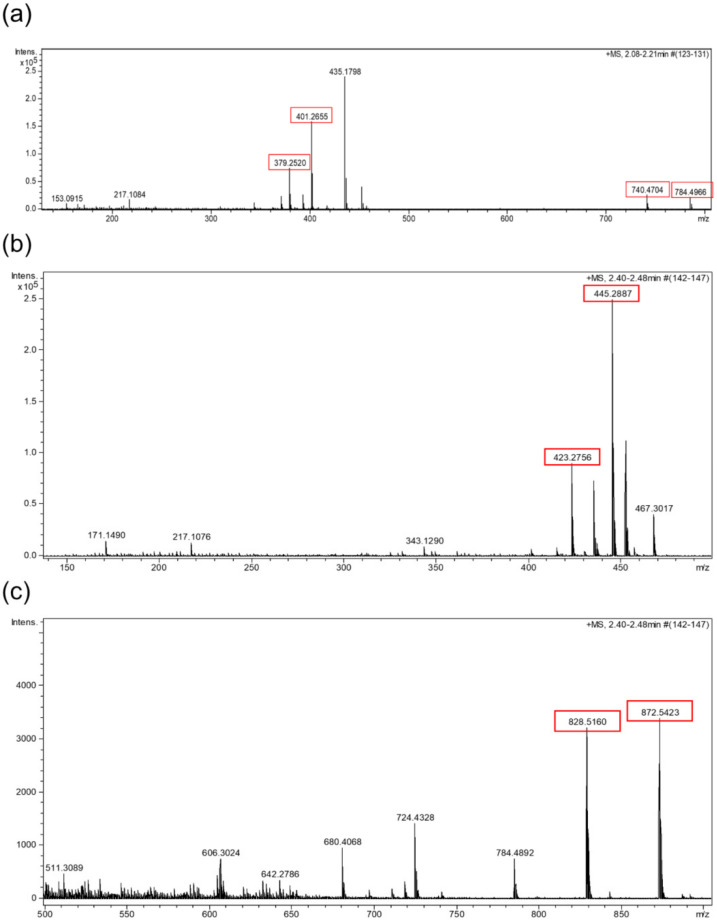
HRMS spectrum expansion of component P1 (**a**). [M + H]^+^ and [M + H + NH_4_]^2+^ adducts of the compounds P1A and P1B are highlighted. P1A m/z values for [M + H]^+^ and [M + H + NH_4_]^2+^ are 740.4704 and 379.2520, respectively. P1B m/z values for [M + H]^+^ and [M + H + NH_4_]^2+^ are 784.4966 and 401.2655, respectively. HRMS spectrum expansions of component P2 (**b**,**c**). [M + H]^+^ and [M + H + NH_4_]^2+^ adducts of the compounds P2A and P2B are highlighted. P2A m/z values for [M + H]^+^ and [M + H + NH_4_]^2+^ are 828.5160 and 423.2756, respectively. P2B m/z values for [M + H]^+^ and [M + H + NH_4_]^2+^ are 872.5423 and 445.2887, respectively.

Four different unknown compounds were detected in fraction C of the CM extract. Component P1 consisted of a mixture of two compounds (P1A and P1B) which displayed the HRMS adducts [M + H]^+^ and [M + H + NH_4_]^2+^ (Figure 7a). Component P2 corresponded to another mixture of two compounds (P2A and P2B), which also displayed HRMS adducts [M + H]^+^ and [M + H + NH_4_]^2+^ (Figure 7b,c). Considering the observed adducts, compounds P1A, P1B, P2A and P2B were assigned the molecular formulae C_39_H_61_N_7_O_7_, C_41_H_65_N_7_O_8_, C_43_H_69_N_7_O_9_ and C_45_H_73_N_7_O_10_, respectively. The best coincidences with these formulae in the Dictionary of Natural Products database (DNP) corresponded to cyclic peptides, but only P1A and P2A had hits in the database. Box jellyfishes are known to be outstanding producers of toxic proteins and peptides which are present in their venoms [54,55], and small cyclic peptides have also been isolated from jellyfish-derived fungi [56]. C_39_H_61_N_7_O_7_ retrieved as a hit the peptide pseudacyclin A in the DNP, while C_43_H_69_N_7_O_9_ retrieved the peptide taxlllaid E [57,58]. Compounds present in fraction C are most likely to be peptides, but further jellyfish sampling and studies are necessary to confirm their identity.

Other compounds present within the fractions of both species were the well-known polyunsaturated fatty acids, eicosapentaenoic acid (EPA) and dodecosahexaenoic acid (DHA). Both compounds were reported to be present in jellyfish extracts according to the literature [58,59,60]. EPA is already known to possess antiproliferative properties. In particular, EPA reduces the cell viability of lung carcinoma cells A549 by 50% after 72 h of treatment at a concentration of 6.05 μM [61]. DHA has also been reported to possess antiproliferative properties. It reduced the viability of breast (MDA-MB-231 and MCF-7), pancreatic (MiaPaca-2) and colorectal (CaCo-2, SW-620) cancer cell lines at concentrations between 10 and 100 µM [62]. Both compounds can therefore be contributing to the biological activity observed when testing the fractions.

Finally, CTOA contained two components with molecular formulae C_18_H_31_NO_2_ and C_22_H_37_NO_2_. These compounds are most likely amide derivatives of polyunsaturated fatty acids. They are reported as natural self-defense agents in plants with several known biological activities [63], but a few of them have been reported in marine natural sources alike [64,65]. This would be the first time this class of compounds is reported in jellyfish. However, further isolation and characterization procedures should be performed in order to confirm their identity and their potential contribution to the bioactivity observed.

## 3. Discussion

This study shows that *C. tuberculata* oral arms and whole *C. marsupialis* extracts have antiproliferative properties against human cell lines and, in particular, melanoma A2058 and lung A549 cells. Results showed that, after fractionation, fraction C was specific against melanoma cells. It has been previously reported that bioactivity is not identical between total extracts and fractions, because of less salt concentration in fractions [66], as well as synergistic effects of components in the total extracts or loss of unstable compounds in fractions. Fraction C of both species was active against melanoma human cells at 10 and 100 µg/mL in both species. Fraction C was enriched in glycolipids and phospholipids, according to the solid phase extraction fractionation procedure by Cutignano et al. [66]. This class of compounds has been previously shown to exert various biological activities, such as anticancer [67] and immunomodulatory [68,69]. These new findings open exciting potential scenarios on the use of jellyfish extracts for cancer treatment or prevention, as possible innovative drugs or food additives.

The biological activity against cancer cells by *C. tuberculata* was unexpected, considering that this scyphomedusa is harmless to humans [31] and there are no toxic or antiproliferative compounds known from this species to date [5]. Conversely, *C. marsupialis* is known to induce painful stings [31] and produces compounds with negative effects on human cells, such as hemolysin, a compound with hemolytic activity, and four cytolysins (i.e., CmHl1, CmHl5, CmH17 and CmNt) [40,41]. However, these biological activities found in compounds extracted from *C. marsupialis* have found little application until now.

In line with its harmlessness, *C. tuberculata* appears to be a promising source of compounds which benefit human health, such as collagen and antioxidant peptides [9]. This Mediterranean scyphomedusa contains the highest amount of collagen of all other scyphomedusae where its content was determined, together with the other Mediterranean rhizostome species, *R. pulmo*, and more than the quantity contained in other scyphomedusae consumed as food in the eastern seas [4]. Additionally, *C. tuberculata* hosts the dinoflagellate endosymbionts *Philozoon medusarum* [70], which produces several compounds through photosynthesis which support the metabolism of the scyphomedusa, although these pathways are not yet well defined [71]. 

In addition to the compounds mentioned above, fraction C of both species, which was the most active, was enriched in fatty acids and fatty acid amide derivatives [66]. Considering the high content in fatty acids (omega 3 and 6), antioxidant, phenolic compounds and proteins, jellyfish have been proposed as a new biomass potentially useful as human food worldwide [9,14]. Overall, our findings suggest that both *C. marsupialis* and *C. tuberculata* may have important applications in the biomedical field, in addition to those described above.

Our results are in line with recent reviews [4,5], which highlighted that jellyfish are an unexploited source of high-value compounds, which are already used or have promising applications in several biotechnological fields, including nutraceutical, cosmeceutical and biomedical sectors. The advantage of using jellyfish as a source of high-value compounds is two-fold. Firstly, jellyfish are abundant and often give rise to blooms which interfere with fisheries and power plant activities along the coasts [7]. Secondly, they are cheap to obtain as a by-catch of the above-mentioned activities and using them for their compounds may actually become a way to recycle their biomass within the context of a circular economy and a sustainable use of resources [72,73,74]. In particular, the extraction of high-value compounds may be an effective way to exploit the resource in western countries, where the consumption of jellyfish as food is extremely limited due to the different dietary tradition compared to eastern countries.

## 4. Materials and Methods

### 4.1. Sample Collection and Processing

Two scyphomedusae (*P. noctiluca* and *R. pulmo*) and the cubomedusa *C. marsupialis* were collected in the Gulf of Naples between 2017 and 2019, while the scyphomedusa *C. tuberculata* was caught in Palinuro during August 2019 (Table 2). All scyphomedusae were collected using a dipnet from the boat, while the cubomedusa was collected directly by SCUBA divers. All specimens were transported to the SZN in plastic buckets filled with seawater from the sampling site. In laboratory, medusae were dissected and frozen at −30 °C. Samples were then freeze-dried to remove the water and concentrate the organic matter.

### 4.2. Extract Preparation

Lyophilized jellyfish were resuspended in methanol (proportion 1:5, *w*/*v*). Samples were vortexed to ensure that methanol completely soaked the freeze-dried jellyfish that were then placed on ice for 30 min, then the obtained suspension was sonicated with 2 short bursts of 30 s each, followed by intervals of 30 s for cooling. Samples were then centrifuged at 3000 rpm for 20 min, and the supernatant was transferred into a rotary evaporator system (Rotavapor). Once a consistent reduction of total volume was reached, samples were evaporated under a nitrogen stream.

### 4.3. Fractionation of the Raw Extract

Fractionation of each extract (an aliquot with a maximum weight of 20 mg) was performed by solid phase extraction (SPE) using Chromabond^®^ HR-X cartridges (6 mL/500 mg) as reported by Cutignano et al. [66]. Briefly, the samples were loaded into the cartridges and then eluted using different mixtures of solvents to get five different fractions: 100% H_2_O (2 mL, discarded); 100% H_2_O (6 mL, fraction A); CH_3_OH/H_2_O (50:50, 9 mL, fraction B); CH_3_CN/H_2_O (70:30, 9 mL, fraction C); 100% CH_3_CN (9 mL, fraction D); CH_2_Cl_2_/CH_3_OH (90:10, 9 mL, fraction E). Each fraction was evaporated under reduced pressure, weighted, and preserved at −20 °C.

### 4.4. Cell Lines

Human hepatocellular liver carcinoma (HepG2; ATCC^®^ HB-8065™) were cultured in EMEM medium, human melanoma cells (A2058; ATCC^®^CRL-11147^TM^) were cultured in DMEM, human keratynocytes (HaCaT, CLS n. 300493) were cultured in DMEM medium, adenocarcinomic human alveolar basal epithelial cells (A549; ATCC^®^CL-185^TM^) were cultured in F-12K medium and human normal lung fibroblasts (MRC-5; ATCC^®^ CCL-171™) were cultured in EMEM medium. The media were supplemented with 10% fetal bovine serum, 50 U·mL^−1^ penicillin and 50 μg·mL^−1^ streptomycin. Human cell lines A2058, A549, MRC-5 and HepG2 were bought at ATCC (https://www.lgcstandards-atcc.org/ accessed on 1 June 2019). The HACAT cells were bought at the CEINGE facility cell bank (https://www.ceinge.unina.it/en/cell-cultures accessed on 1 June 2019).

### 4.5. In Vitro Antiproliferative Assays

Human tumor cell lines (HepG2, A2058 and A549) and the normal cell lines (MRC5 and HaCaT) were seeded in 96-well microtiter plates at a density of 1 × 10^4^ cells/well and incubated at 37 °C to allow cell adhesion to the plates. After 16 h, the medium was replaced with fresh medium containing increasing concentrations of extracts or fractions (100, 10, 1, 0.1 and 0.01 μg·mL^−1^) for 24, 48 or 72 h. Extracts and fractions were dissolved in dimethyl sulfoxide (DMSO) with the maximum concentration for DMSO used of 1% (*v*/*v*). Each concentration was tested at least in triplicates. After 72 h, 3-(4,5-dimethyl-2-thizolyl)-2,5-diphenyl-2H-tetrazolium bromide (MTT; A2231,0001, AppliChem Panreac Tischkalender, Darmstadt, GmbH) was added. Briefly, the medium was replaced with a medium containing MTT at 0.5 mg·mL^−1^ and plates were incubated for 3 h at 37 °C. After incubation, cells were treated with isopropyl alcohol for 30 min at room temperature to dissolve MTT. Absorbance was measured at OD = 570 nm using a microplate reader (Multiskan™ FC Microplate Photometer, Thermo Fisher Scientific, Waltham, MA, USA). Cell proliferation was expressed as a percentage of cell proliferation in the presence of the tested samples, with respect to untreated control cultures (with only DMSO).

### 4.6. RNA Extraction and Reverse Transcription-Quantitative Polymerase Chain Reaction (RT-qPCR)

A2058 cells were seeded in 6-well plates (400,000 per well) and left 16 h for attachment. The seeded cells were then treated in the presence of fraction C of both CM and CTOA samples at 1 μg·mL^−1^ for 48 h at 37 °C. Cells were washed by adding phosphate buffered saline (PBS 1X). Cells were lysed by adding 1 mL of Trisure Reagent (Meridian bioscience, Memphis, TN, USA). RNA was isolated as previously described [75]. RNA concentration, quality, and purity were assessed using an ND-1000 UV-Vis spectrophotometer (NanoDrop Technologies, Thermo Fisher Scientific, Waltham, MA, USA), monitoring the absorbance at 260 nm, and the 260/280 nm and 260/230 nm ratios (both ratios were about 2.0). RNA quality was evaluated by gel electrophoresis that showed intact RNA. About 500 ng of RNA underwent a reverse transcription reaction using the RT2 first strand kit (cat. 330401, Qiagen, Hilden, Germany) according to the manufacturer’s instructions. The RT-qPCR analysis was performed in duplicate using the RT2 Profiler PCR Array kit (cat. PAHS-212ZE-4, Qiagen, Hilden, Germany) to analyze the expression of 84 genes involved in cell death signaling pathways. Plates were run on a ViiA7 (Applied Biosystems, Foster City, CA, USA, 384-well blocks). The PCR program consisted of a denaturation step at 95 °C for 20 s followed by 40 cycles at 95 °C for 15 s and 60 °C for 1 min. The cycle threshold (Ct)-values were analyzed with PCR array data analysis online software (GeneGlobe Data Analysis Center at https://geneglobe.qiagen.com/us/analyze, accessed on 10 September 2021, Qiagen, Hilden, Germany). Real-time data were expressed as the fold of expression, describing the changes in gene expression between cells treated in the presence fraction C of CM and cells treated in the presence of DMSO alone (control). The PCR array data analysis software uses Student’s *t*-test for statistical analysis. Only expression values greater than a two-fold difference with respect to the controls were considered significant.

### 4.7. Dereplication

Fractions were analyzed by HPLC-UV-HRESIMS on an Agilent 1200 RR coupled to a Bruker maXis QToF spectrometer with electrospray ionization, as reported by [76]. Data were analyzed following the guidelines developed by Fundación MEDINA [77] and compared with the data available on the Fundación MEDINA internal database and the Dictionary of Natural Products and Reaxys databases. The identity of the components was putatively assigned by comparison of their HRMS spectra, UV maxima, retention time, natural source and biological activity with the data available in DNP (Dictionary of natural products) and Reaxys (“Others; Isolated from natural source” filter).

### 4.8. Statistics

Arithmetic means ± the standard deviations (SD) were calculated and compared by a two-tailed Student *t*-test. Differences at *p* < 0.05 were regarded as significant.

## Figures and Tables

**Table 1:** Transcriptional modulation of genes involved in human cell death signaling pathways in treated A2058 cells. Gene transcription is not considered affected by compound treatment if fold regulation is in the range ± 2.

UniGEne	RefSeq	Symbol	Description	Fold	SD
Genes up-regulated by CTOA fraction C					
Hs.552567	NM_001160	APAF1	Apoptotic peptidase activating factor 1	2.77	0.245
**Genes down-regulated by CTOA fraction C**					
Hs.592068	NM_020655	JPH3	Junctophilin 3	−2.54	0.792
Hs.241570	NM_000594	TNF	Tumor necrosis factor	−2.22	0.227
Hs.654567	NM_005848	DENND4A	DENN/MADD domain containing 4A	−2.16	0.019
**Genes up-regulated by CM fraction C**					
Hs.442337	NM_176823	S100A7A	S100 calcium binding protein A7A	4.28	0.917
Hs.552567	NM_001160	APAF1	Apoptotic peptidase activating factor 1	4.21	0.079
Hs.716464	NM_003766	BECN1	Beclin 1	2.65	0.056
Hs.578973	NM_015247	CYLD	Cylindromatosis (turban tumor syndrome)	2.20	0.428
Hs.181301	NM_004079	CTSS	Cathepsin S	2.04	0.141
**Genes down-regulated by CM fraction C**					
Hs.356076	NM_001167	XIAP	X-linked inhibitor of apoptosis	−149.92	9.751
Hs.513667	NM_003946	NOL3	Nucleolar protein 3 (apoptosis repressor with CARD domain)	−19.25	4.862
Hs.87236	NM_012188	FOXI1	Forkhead box I1	−6.87	1.369
Hs.654567	NM_005848	DENND4A	DENN/MADD domain containing 4A	−6.14	0.416
Hs.592068	NM_020655	JPH3	Junctophilin 3	−2.79	0.292
Hs.696238	NM_001166	BIRC2	Baculoviral IAP repeat containing 2	−2.26	0.553
Hs.592244	NM_000074	CD40LG	CD40 ligand	−2.12	0.137

**Table 2:** The species names, taxonomy, collection site and date of the specimens used in this study.

Taxon	Species	Collection Site	Collection Date
Cubomedusae	*Carybdea marsupialis*	Gulf of Naples	23/10/2018
Scyphomedusae	*Pelagia noctiluca*	Gulf of Naples	15/05/2019
	*Rhizostoma pulmo*	Gulf of Naples	27/10/2017
	*Cotylorhiza* *tuberculata*	Palinuro	26/08/2019

## Data Availability

Not applicable.
